# A Neuromorphic Device Implemented on a Salmon‐DNA Electrolyte and its Application to Artificial Neural Networks

**DOI:** 10.1002/advs.201901265

**Published:** 2019-07-15

**Authors:** Dong‐Ho Kang, Jeong‐Hoon Kim, Seyong Oh, Hyung‐Youl Park, Sreekantha Reddy Dugasani, Beom‐Seok Kang, Changhwan Choi, Rino Choi, Sungjoo Lee, Sung Ha Park, Keun Heo, Jin‐Hong Park

**Affiliations:** ^1^ Department of Electrical and Computer Engineering Sungkyunkwan University Suwon 16419 Korea; ^2^ School of Electrical and Electronic Engineering Nanyang Technological University 50 Nanyang Avenue 639798 Singapore Singapore; ^3^ Department of Physics Sungkyunkwan University Suwon 440‐746 South Korea; ^4^ Division of Materials Science and Engineering Hanyang University Seoul 133–791 South Korea; ^5^ Material Science and Engineering Inha University Incheon 402–751 South Korea; ^6^ SKKU Advanced Institute of Nanotechnology (SAINT) Sungkyunkwan University Suwon 440–746 South Korea

**Keywords:** handwritten digit pattern recognition, neural devices, neuromorphic devices, salmon DNA, synaptic devices

## Abstract

A bioinspired neuromorphic device operating as synapse and neuron simultaneously, which is fabricated on an electrolyte based on Cu^2+^‐doped salmon deoxyribonucleic acid (S‐DNA) is reported. Owing to the slow Cu^2+^ diffusion through the base pairing sites in the S‐DNA electrolyte, the synaptic operation of the S‐DNA device features special long‐term plasticity with negative and positive nonlinearity values for potentiation and depression (α_p_ and α_d_), respectively, which consequently improves the learning/recognition efficiency of S‐DNA‐based neural networks. Furthermore, the representative neuronal operation, “integrate‐and‐fire,” is successfully emulated in this device by adjusting the duration time of the input voltage stimulus. In particular, by applying a Cu^2+^ doping technique to the S‐DNA neuromorphic device, the characteristics for synaptic weight updating are enhanced (|α_p_|: 31→20, |α_d_|: 11→18, weight update margin: 33→287 nS) and also the threshold conditions for neuronal firing (amplitude and number of stimulus pulses) are modulated. The improved synaptic characteristics consequently increase the Modified National Institute of Standards and Technology (MNIST) pattern recognition rate from 38% to 44% (single‐layer perceptron model) and from 89.42% to 91.61% (multilayer perceptron model). This neuromorphic device technology based on S‐DNA is expected to contribute to the successful implementation of a future neuromorphic system that simultaneously satisfies high integration density and remarkable recognition accuracy.

In the emerging “Big Data” era, it is challenging to handle large amounts of informal data, such as characters, images, sounds, and other unstructured data formats using current computing technology based on von Neumann architecture (series and linear processing).^1^, ^2^, ^3^ Under this technical circumstance, a neuromorphic computing technology featuring parallel and nonlinear processing has been recently proposed to achieve an efficient computing platform for informal data.^4^, ^5^ Neuromorphic computing is performed on the basis of a hardware neural network (HNN),^6^, ^7^, ^8^ which conceptually mimics a biological neural network consisting of synapses and neurons.^9^, ^10^ Accordingly, many studies aimed at implementing the HNN have been attempted by fabricating artificial synapse and neuron device arrays.^11^, ^12^, ^13^ Although complementary metal‐oxide semiconductor (CMOS) circuit techniques have achieved synaptic and neural functions,^12^, ^14^, ^15^ a large number of CMOS transistors are required for the synapse and neuron circuits (10 transistors per synapse^16^ and 5 transistors per neuron^17^). However, when such a large number of devices are integrated into an HNN system, they will cause significant problems such as high power consumption and low synapse/neuron packing density.^1^, ^10^, ^18^, ^19^ Therefore, it is essential to develop synaptic and neural devices that are highly efficient in energy consumption and can be integrated at a high density.

Since Jo et al.^20^ reported artificial synaptic behaviors, such as long‐term potentiation/depression (LTP/LTD), in a Si/Ag‐based memristor device in 2010, two‐terminal memristive devices, such as phase‐change random‐access memory (PCRAM),^16^, ^21^, ^22^ resistive‐switching random‐access memory (ReRAM),^23^, ^24^, ^25^ and conductive bridge random‐access memory (CBRAM) devices^26^, ^27^ have been studied actively as promising synaptic and neural devices for HNNs. Such devices could gradually change the conductance of the path of current according to input voltage pulses,^16^, ^21^, ^22^, ^23^, ^24^, ^25^, ^26^, ^27^ thereby allowing for the implementation of the functional operation of a synapse within a unit device. In particular, studies on memristive synapses have demonstrated that high‐density HNNs can be constructed via fabrication in a crossbar point array structure.^28^, ^29^ Recently, HfO*_x_*‐,^30^ TaO*_x_*‐,^31^, ^32^ or TiO*_x_*‐33, 34 based ReRAM devices and Ge_2_Sb_2_Te_5_‐16, 35, 36 or Mott‐insulator‐37 based PCRAM devices have successfully emulated synaptic dynamics, such as LTP/LTD characteristics and excitatory/inhibitory postsynaptic currents (EPSC/IPSC). Prezioso et al.^38^ fabricated a neural network based on an Al_2_O_3_/TiO_2_ *_x_* memristor crossbar point array and demonstrated successful pattern classification of 3 × 3 binary images. In addition, PCRAM devices could emulate firing and relaxation functionalities of biological neurons owing to easy transition between insulator and metal. Tuma et al.^39^ and Adda et al.^40^ demonstrated a PCRAM‐based neuron device with a basic neural function “integrate and fire,” subsequently presenting the possibility of simplifying the neuronal circuit to a unit device level. Very recently, a few studies that explore implementation of both synaptic and neuronal operations in one device have been reported.^35^, ^36^, ^39^, ^40^ In particular, Wang et al.^41^ successfully built a fully memristive neural network through the integration of diffusive memristor (Ag/SiO_2_:Ag/Ag) neurons and drift memristor (Ta/HfO*_x_*/Pd) synapses, where they then demonstrated an unsupervised synaptic weight updating and a pattern classification by their fully memristive neural network.

Herein, we report a bio‐inspired neuromorphic device operating as synapse and neuron simultaneously that is fabricated on a Cu^2+^‐doped salmon deoxyribonucleic acid (S‐DNA) electrolyte. Owing to the slow Cu^2+^ diffusion through the base pairing sites in the S‐DNA, the S‐DNA device presents the special synaptic characteristic (LTP characteristic with negative nonlinearity), which consequently improves the accuracy of numerical digit pattern recognition of S‐DNA‐based neural network. In addition, this S‐DNA device successfully emulates the integrate‐and‐fire behavior of a neuron. As the S‐DNA synaptic and neural operations are based on Cu redox reactions (Cu ↔ Cu^2+^ + 2 e^−^), we discuss how the Cu^2+^ doping applied to the S‐DNA electrolyte influences the synaptic and neural operating characteristics, respectively, in terms of: i) the nonlinearity and weight update margin of LTP/LTD characteristics and ii) the threshold condition of firing phenomenon. We then predict the accuracy of MNIST pattern recognition with respect to the number of learning steps, where a single‐layer ANN structure (or a multilayer ANN structure for +NeuroSim simulator) and a back‐propagation weight update algorithm are applied.

In a biological synapse, neurotransmitters are released from a presynaptic neuron. They then diffuse toward a postsynaptic neuron when an active signal reaches the presynaptic neuron. Subsequently, the neurotransmitters are combined with receptors at the postsynaptic neuron, generating a postsynaptic signal.^42^, ^43^, ^44^ As shown in **Figure** **1**a, the signal transportation mechanism of the S‐DNA device resembles that of the biological synapse. When a presynaptic spike signal (*V*_pre_) is applied to the Cu/Ti electrode, an electric‐field‐assisted diffusion of Cu^2+^ occurs between Cu/Ti and Pt electrodes. The diffused Cu^2+^ adjust the conductivity of the S‐DNA device (synapse weight) by contributing to the formation of a Cu filament. Here, a Ti buffer layer was inserted at the Cu/S‐DNA interface to improve the operating stability of the S‐DNA device.^45^ The buffer layer is expected to reduce the: i) Cu^2+^ diffusion rate into the S‐DNA and ii) activation energy for the formation of the Cu filament. To understand the diffusion mechanism of Cu^2+^ in the S‐DNA electrolyte in detail, we performed Raman spectroscopy analysis and Fourier‐transform infrared spectroscopy (FT‐IR) analyses. Figure 1b shows the Raman spectra measured on Cu^2+^‐doped S‐DNA films with various concentrations (0, 0.1, and 1 × 10^−3^ m). In the spectral range from 600 to 1400 cm^−1^, we observed eight Raman peaks. Here, the four Raman peaks between 786 and 1095 cm^−1^ indicate the vibration mode of the backbone of S‐DNA,^46^, ^47^ and other Raman peaks (from 1248 to 1375 cm^−1^) are related to the S‐DNA base pairs of cytosine (C), adenine (A), thymine (T), and guanine (G).^46^, ^47^ The Raman peak intensities monotonically decreased with increasing Cu^2+^ concentrations. In particular, the intensity decrease in the Raman peaks for the base pairs was more severe than that for backbone peaks. As the Cu^2+^ concentration increased from 0 to 1 × 10^−3^ m, the Raman peak intensities related to the S‐DNA backbone and base pairs decreased by ≈18% and 40%, respectively, as shown in Figure 1c.

**Figure 1:**
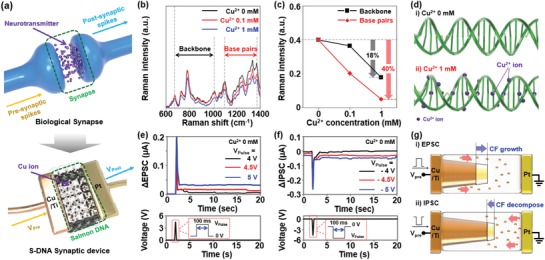
a) Schematic of biological synapse (top) and S‐DNA synaptic device (bottom). b) Raman spectra of Cu^2+^‐doped S‐DNA films with various concentrations (0, 0.1, and 1 × 10^−3^ m), and c) Raman intensities extracted from the spectra. d) Graphical illustration presenting the intercalation of Cu^2+^ in the 0 × 10^−3^ m (up) and 1 × 10^−3^ m (down) Cu^2+^‐doped S‐DNA molecules. e) PSC response of S‐DNA device induced by an excitatory voltage pulse with magnitudes of 4 V (black), 4.5 V (red), or 5 V (blue). f) PSC response of S‐DNA synaptic device induced by an inhibitory voltage pulse with magnitudes of −4 V (black), −4.5 V (red), or −5 V (blue). Here, the duration time (*t*_d_) of the excitatory/inhibitory voltage pulses was 100 ms. g) Illustration showing conductive filament growth (up)/decomposition (down) by excitatory/inhibitory voltage pulses.

Similarly, in the FT‐IR analysis shown in Figure S3 in the Supporting Information, we confirmed that the FT‐IR peak intensities related to the S‐DNA base pairs were reduced by Cu^2+^ doping. This is because Cu^2+^ ions are preferably bound at the base pairing sites, thereby influencing the S‐DNA molecular structure.^48^ The binding of Cu^2+^ to the S‐DNA molecule is graphically illustrated in Figure 1d. These Cu^2+^ ions are, thus, expected to move through the base pairs in the S‐DNA molecule, enabling conductive filament‐based switching. We then investigated the postsynaptic current characteristics of the S‐DNA device to confirm its feasibility as a synaptic device. Figure 1e,f shows postsynaptic current change (ΔPSC) in the undoped S‐DNA device, where we applied excitatory (*V*_pre_ = 4, 4.5, and 5 V) and inhibitory (*V*_pre_ = −4, −4.5, and −5 V) pulses with a duration time (*t*_d_) of 100 ms, respectively. The amplitude of the excitatory and inhibitory pulses was determined by the following procedure: i) we experimentally confirmed the set and reset voltages (*V*_SET_ and *V*_RESET_) of the S‐DNA devices (Figure S4, Supporting Information); ii) we then selected three different pulse amplitudes near the *V*_SET_ and *V*_RESET_ values. Here, the chosen amplitudes for the excitatory pulses were 4, 4.5, and 5 V, and the ones for the inhibitory pulses were −4, −4.5, and −5 V because the *V*_SET_ and *V*_RESET_ were 4.5 and −4.5 V, respectively. The excitatory postsynaptic current (EPSC) value increased to 7.2 nA after applying the +4 V pulse, whereas the inhibitory postsynaptic current (IPSC) induced by the −4 V pulse decreased to −2.7 nA. Moreover, the EPSC and IPSC values could be controlled by adjusting the pulse amplitude. When increasing the amplitude of the excitatory pulse from 4 to 5 V, the EPSC value varied from 7.2 to 37.1 nA. Similarly, the ΔIPSC value decreased from −2.7 to −46.6 nA when the amplitude of the inhibitory pulse reduced to −5 V. The EPSC and IPSC characteristics of the S‐DNA device can be explained by the growth and decomposition of Cu filament by the presynaptic spike signal (*V*_pre_), respectively, as illustrated in Figure 1g. When an excitatory *V*_pre_ is applied to the Cu/Ti electrode, Cu^2+^ ions diffuse into the S‐DNA electrolyte and then a Cu filament starts growing from the Cu surface through the reduction process (Cu^2+^ + 2e^−^ → Cu). In contrast, an inhibitory *V*_pre_ will lead to the oxidation of the Cu filament (Cu → Cu^2+^ + 2e^−^), resulting in the decomposition of the filament in the S‐DNA film.

The S‐DNA device was investigated in more detail in terms of its long‐term synaptic plasticity, such as its LTP/LTD characteristics. **Figure** **2**a presents the LTP/LTD characteristics of the S‐DNA device, to which we applied a series of 15 excitatory/inhibitory pulses (*V*_pre_ = 4.5 V/−4.5 V and *t*_d_ = 100 ms). Here, the amplitudes of the excitatory and inhibitory pulses were chosen on the basis of the set and reset voltages of the S‐DNA device. As the excitatory pulses were applied, the current level was potentiated gradually from 1.7 to 52.1 nA. After that, the current level was depressed by the inhibitory pulses, eventually recovering to its initial level. From the LTP/LTD characteristic curves shown in Figure 2b, we extracted nonlinearity factors (α) and weight update margins (Δ*G* = *G*_max_ − *G*_min_). Here, the blue dotted lines indicate ideal LTP/LTD curves (α = 1) and the circle symbol shows the experimental conductance data. The conductance values were fitted by using the equations *G* = *B*(1−*e* ^P/A^) + *G*_min_ and *G* = −*B*(1−*e* ^(^ *^P^* ^−^ *^P^* ^max)/A^) + *G*_min_, where *B* is a fitting factor, *P* is the pulse number, and *A* is the nonlinearity of potentiation (α_p_) and depression (α_d_). After fitting the data, we confirmed a special potentiation characteristic with a negative nonlinearity value in the S‐DNA device. Typical synaptic devices have an LTP curve with a positive nonlinearity value (e.g., ReRAM and PCRAM).^21^, ^22^, ^23^, ^24^, ^25^, ^26^, ^27^, ^28^ The extraordinary LTP/LTD profiles with opposite nonlinearity values (negative α_p_ and positive α_d_) can be explained by the harsh Cu^2+^ diffusion condition in the S‐DNA electrolyte owing to the relatively lower number of defective sites. For easier understanding, we illustrated two operating mechanisms for the S‐DNA device, as shown in Figure 2c. When the 1st to 7th excitatory pulses are transmitted (Case I in LTP), more Cu^2+^ ions from the Cu electrode diffuse through the base pairing sites of the S‐DNA to the Pt electrode and subsequently increase the conductance of the S‐DNA device.^49^, ^50^ Here, owing to the slow diffusion of the Cu^2+^ in the S‐DNA electrolyte, the conductance value is predicted to slowly increase until the Cu^2+^ reacted with the electrons coming from the Pt‐side and a Cu filament is subsequently formed on the Cu‐side.^50^ After forming a Cu filament partially on the Cu‐side (Case II in LTP), the injected Cu^2+^ ions contribute not only to an increase in the conductance of the S‐DNA electrolyte but also to further growth of the Cu filament,^50^ thereby increasing the total conductance value more rapidly. This contribution of the Cu filament to the conductance change therefore provides the negative LTP nonlinearity (α_p_ < 0). When a series of inhibitory pulses (from 1st to 7th) is applied, a Joule heating–assisted oxidation process (Cu → Cu^2+^ + 2e^−^) causes the dissolution of the Cu filament, resulting in an exponential decrease in conductance (Case II in LTD).^49^, ^50^ After the Cu filament completely disappears, the inhibitory pulse induces the diffusion of Cu^2+^ toward the Cu‐side (Case I in LTD) and the diffused Cu^2+^ ions are trapped at the Cu/S‐DNA interface.^49^ This reduces the amount of Cu^2+^ in the S‐DNA electrolyte gradually as an inhibitory pulse is supplied, finally leading to a linear decrease in conductance. In particular, the magnitude of the nonlinearity factors for the LTP and LTD curves varied oppositely when the S‐DNA electrolyte was doped with a higher Cu^2+^ concentration. As shown in Figure 2d, |α_p_| reduced as the Cu^2+^ concentration increased from 0 to 1 × 10^−3^ m (|α_p_|: 31 → 20), whereas |α_d_| increased from 12 to 18. This can be explained by the increase of the Cu^2+^ concentration in the S‐DNA electrolyte. Before the formation of the Cu filament (LTP‐side blue region in Figure 2e), the conductance change is only determined by the amount of Cu^2+^ ions injected from the Cu electrode. Thus, the conductance is not that different before/after Cu^2+^ doping (undoped: 6.2 nS, 1 × 10^−3^ m Cu^2+^‐doped: 18.4 nS). However, after the Cu filament partially forms, additionally supplied Cu^2+^ ions are predicted to help the growth of the Cu filaments (LTP‐side red region).^50^ These Cu^2+^ ions thicken the Cu filament, unlike in the control S‐DNA device, resulting in a large conductance change (undoped: 27.7 nS, 1 × 10^−3^ m Cu^2+^‐doped: 268.6 nS). Similar conductance change is expected in the LTD‐side two regions with (red) or without (blue) a Cu filament. Consequently, the 1 × 10^−3^ m Cu^2+^‐doped S‐DNA device showed favorably symmetric LTP/LTD characteristics (α_p_: −20/α_d_: 18). The weight update margin (Δ*G*) was also dependent on the Cu^2+^ doping concentration. By increasing the Cu^2+^ concentration from 0 to 1 × 10^−3^ m, Δ*G* was improved by factors of 8.2 (Δ*G*: 33 → 287 nS), as seen in Figure 2f.

**Figure 2:**
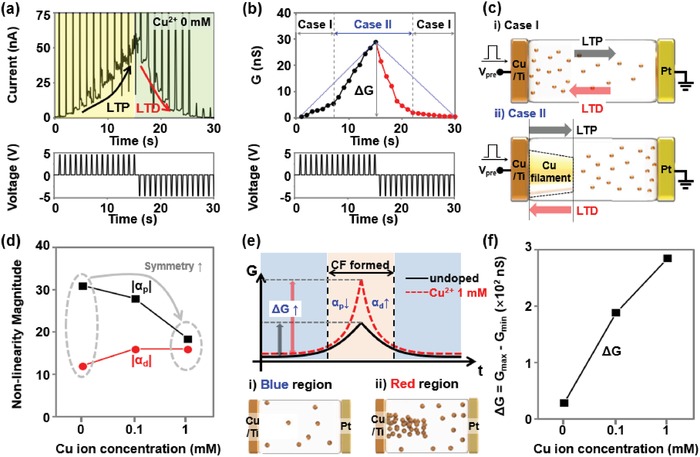
a) Current and b) conductance values of the undoped S‐DNA device as a function of time, where 15 excitatory/inhibitory voltage pulses (*V*_pre_ = ± 4.5 V, *f* = 1 Hz, and *t*_d_ = 100 ms) were continuously applied to investigate the LTP/LTD characteristics. c) Schematics of Cu^2+^ diffusion (Case I) and conduction filament growth/decomposition (Case II) in the S‐DNA device due to applying presynaptic voltage pulses. d) Magnitude of nonlinearity for LTP (α_p_) and LTD (α_d_) as a function of Cu^2+^ concentration (0, 0.1, and 1 × 10^−3^ m). e) LTP/LTD profiles of undoped/Cu^2+^‐doped S‐DNA devices and a schematic showing the S‐DNA before (blue region) and after (red region) the formation of conduction filaments. f) Weight update margin in the LTP/LTD curves as a function of Cu^2+^ concentration (0, 0.1, and 1 × 10^−3^ m).

We also found that the operating mode of the S‐DNA device can convert from synapse to neuron by adjusting the duration time (*t*_d_) of voltage stimulus pulse. **Figure** **3**a shows the current of the S‐DNA device as a function of time, where a positive voltage pulse was applied repeatedly (*V*_pre_ = 4.5 V, *f* = 1 Hz). Like in the previous case in which a short voltage pulse with *t*_d_ = 50 ms was applied, the current of the S‐DNA device gradually increased as pulses with *t*_d_ = 100 ms were supplied continuously (*I* = 157 nA after applying the tenth pulse), representing long term plasticity. The increment in the current became larger when the duration time increased to 500 ms (*I* = 1.19 µA after applying the seventh pulse), and most of the increased current disappeared after the eighth pulse. This phenomenon indicates that the S‐DNA device can operate as a neuron on the basis of two mechanisms: i) “integrating” electrical input signals and ii) “firing” electrical output signals when the integrated value exceeds a threshold point. This device‐mode conversion from synapse to neuron was possible by virtue of the difference in growth rate of the Cu filament, which was dependent on the *t*_d_ of an input pulse. During the operation of the S‐DNA device, the amount of diffused Cu^2+^ ions is proportional to the energy (*V*_pre_ × *t*_d_), which is related to the applied voltage pulse.^51^ When a lower‐energy voltage pulse was applied (*t*_d_ = 100 ms), the Cu filament grew slowly owing to the decreased supply of Cu^2+^, resulting in a small increase in the current (refer to the left panel of Figure 3b). Conversely, in the case of a higher‐energy pulse (*t*_d_ = 500 ms), a higher supply of Cu^2+^ accelerated the growth of the Cu filament. This rapidly grown Cu filament reached the Cu electrode, suddenly generating a spiking current (right panel of Figure 3b). However, this imperfect Cu filament spontaneously dissolved and the conductance of the S‐DNA device returned to its initial level. This current firing phenomenon was more apparent with increasing Cu^2+^ concentration in the S‐DNA electrolyte. Figure 3c presents the electrical characteristics of S‐DNA devices doped with solutions with different Cu^2+^ concentrations (0, 0.1, and 1 × 10^−3^ m). Here, the positive voltage pulses with amplitudes of 4.5, 1.5, and 1 V, which were the same as the EPSC pulses used in the Figure 2 and Figure S4 in the Supporting Information, were applied to the 0, 0.1, and 1 × 10^−3^ m Cu^2+^‐doped S‐DNA devices, respectively. The threshold current level for firing was set as 0.5 µA. In the control device (Cu^2+^ 0 × 10^−3^ m), an output current signal was fired after a +4.5 V voltage pulse was supplied eight times (*N* = 8), where the peak current at the firing state was ≈1 µA. After doping the S‐DNA device with 0.1 × 10^−3^ m Cu^2+^, the output current signal was fired early after the sixth input pulse (*N* = 6), showing a higher peak current of 1.5 µA. The peak current in firing increased up to 1.8 µA and the number of voltage pulses required for firing reduced to *N* = 3 by doping the device with 1 × 10^−3^ m of Cu^2+^. This is attributed to the reduction in activation energy required for neuron firing owing to an increase in Cu^2+^ concentration. Figure 3d describes the neuron operation of the S‐DNA device in the 0 × 10^−3^ m (control) and 1 × 10^−3^ m Cu^2+^ doping conditions. When presynaptic voltage pulses are supplied continuously, Cu ions steadily stick to the Cu filament (pulse integration), eventually generating a spiking current pulse instantly (firing of neuron). From the neuron operating point of view, Cu^2+^ doping allowed for a relatively low amplitude of presynaptic voltage pulse (*V*_0mM_ > *V*_1mM_) and a low number of pulses for neuron firing (*N*_0mM_ > *N*_1mM_). Here, after the initial firing occurred, the S‐DNA neuronal device did not show the firing phenomenon again. This is likely due to the nonvolatile characteristic of Cu conduction filament formed in S‐DNA electrolyte. To remedy this shortcoming, it is necessary to initialize the Cu conductive filament with the assistance of external electrical element or circuit. For instance, Wang et al.^41^ suggested a combination of a diffusive memristor (Ag/SiO_2_:Ag/Ag) and an external electrical component (e.g., capacitor) to achieve a leaky integrate‐and‐fire behavior and a persistent firing of neuron device.

**Figure 3:**
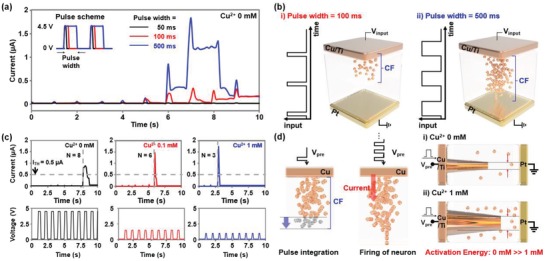
a) Current–time graph of the undoped S‐DNA device with the application of a series of potentiation voltage pulses (*V*_pre_ = 4.5 V, *f* = 1 Hz) with various duration times of 50 (black), 100 (red), and 500 ms (blue). b) Schematic showing conducting filament formation according to different pulse duration times: *t*_d_ = 100 ms (left) and *t*_d_ = 500 ms (right). c) Current–time graphs of 0 × 10^−3^ m (left), 0.1 × 10^−3^ m (middle), and 1 × 10^−3^ m (right) Cu^2+^‐doped S‐DNA devices with the application of a series of potentiation voltage pulses (*V*_pre_ = 4.5, 1.5, and 1 V for 0, 0.1, and 1 × 10^−3^ m Cu^2+^‐doped S‐DNA devices, respectively). Here, the frequency and duration time of the voltage pulses were 1 Hz and 100 ms, respectively. d) Schematic showing the operating mechanism of the S‐DNA neuron device (left) and the conducting filament formation in the S‐DNA neuron devices fabricated on 0 × 10^−3^ m(right top) and 1 × 10^−3^ m (right bottom) Cu^2+^‐doped electrolytes.

With the S‐DNA synaptic devices, we virtually constructed an online learning platform based on single‐ and multilayer artificial neural networks (ANNs), as shown in **Figure** **4**a. For the pattern recognition simulation, we used the MNIST handwritten image dataset containing: i) 60 000 learning images and ii) 10 000 testing images that do not overlap with the learning dataset. The input and output layers of the platform were configured with 784 presynaptic neurons matching with 28 × 28 array pixels of the MNIST images and 10 postsynaptic neurons corresponding to numbers “0” to “9.” Thus, 7840 pairs of potentiation and depression synapses (G^+^ and G^−^) were connected between the presynaptic and postsynaptic neurons; we calculated synaptic weight as *W* = G^+^ − G^−^. After the MNIST image data (*V*_1_−*V*_784_, V) were transmitted to the input layer, they generated the postsynaptic current vector (*I* = ∑*V* × *W*), which is a product of the image data vector and the synaptic weight vector (*W*_1,1_−*W*_784,10_, W). This postsynaptic current vector was then converted into an output vector (*f*_(net)_) through the sigmoid activation function. Finally, to update the synaptic weight, we calculated the difference (δ) between the output value of each output neuron (*V*_out_) and the label data of the MNIST dataset (*V*_label_). If the sign of the product of the δ value and the input value (sgn(δ × *V*_i_)) was positive, the relevant synapses' weights were updated in an increasing direction and vice versa. In the case of δ = 0, the synaptic weight did not change (sgn(δ × *V*_i_) = 0). The MNIST learning phase is described in more detail in Figure S6 in the Supporting Information. The mapping images of 784 synaptic weights for the number “5” are plotted in Figure 4b. We assumed that the mapping image had random weight values before the learning step. After learning the MNIST datasets completely, 784 pixels indicating synaptic weights presented a clear shape of the number “5” that was similar to the target image. Here, the filled pixels in the mapping image indicate that the corresponding synaptic devices have high weight values and vice versa. With an increase in the Cu^2+^ doping concentration, the shape of the number “5” appeared more clearly in the mapping image, in which the color of the filled pixels became darker. This means that the Cu^2+^ doping process successfully improved the learning performance of ANNs consisting of S‐DNA devices. This improvement was possible owing to the negative LTP nonlinearity (thereby, the symmetric LTP/LTD characteristics) and the high weight update margin, which were achieved by the Cu^2+^ doping. To analyze the performance of ANNs quantitatively with respect to various Cu^2+^ doping concentrations, we performed a supervised learning with the MNIST learning dataset and then predicted the recognition rate. The MNIST test phase is detailed in Figure S7 in the Supporting Information. The average recognition rate for every 5000 learning phases was plotted in Figure 4c for cases of 0, 0.1, and 1 × 10^−3^ m Cu^2+^ doping. As predicted, the recognition rate after 60 000 learning phases increased from 38% to 44% as the concentration of Cu^2+^ increased from 0 to 1 × 10^−3^ m. This improvement in recognition rate achieved by increasing Cu^2+^ concentration was also identified by the multilayer perceptron (MLP) model‐based MNIST simulation. Here, the MLP‐based MNIST simulation was conducted on the “+NeuroSim” platform,^52^ which was designed on the basis of a three‐layer perceptron model with 400 input neurons, 100 hidden layer neurons, and 10 output neurons. As shown in Figure S8 in the Supporting Information, the recognition rate after 60 000 learning phases improved from 89.42% to 91.61% as the Cu^2+^ concentration increased from 0 to 1 × 10^−3^ m. The maximum recognition rate in this study (91.61%) was higher than the values obtained by conventional ReRAM‐based ANNs (10–73%), which was also comparable to the value predicted by the FeFET synaptic device (≈90%).

**Figure 4:**
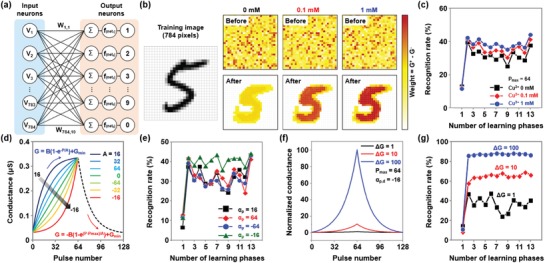
a) Online learning platform using single‐layer perceptron‐based ANN for MNIST pattern recognition. b) The weight mapping images of 784 synaptic weights for number “5” in three cases: 0, 0.1, and 1 × 10^−3^ m Cu^2+^‐doped S‐DNA. c) Average recognition rates versus the number of learning phases for the three cases. d) LTP curves with various LTP nonlinearity values between −16 and 16, in which the following parameters were fixed: LTD nonlinearity (−16), number of states (*P*_max_ = 64), and synaptic weight margin (Δ*G* = 10). e) Average recognition rates versus the number of learning phases for cases with α_p_ = 16 (black), 64 (red), −64 (blue), and −16 (green). f) LTP/LTD curves with different magnitudes of Δ*G*: 1 (black), 10 (red), and 100 (blue). Here, the following parameters were fixed: LTP/LTD nonlinearity (α_p_ = −16, α_d_ = −16), number of states (*P*_max_ = 64), and on/off‐current ratio (*I*_on_/*I*_off_ = 10) g) Average recognition rates versus number of learning phases for cases of Δ*G* = 1 (black), 10 (red), and 100 (blue).

We then investigated how the LTP nonlinearity (α_p_) and the weight update margin (Δ*G*) affect the recognition rate. The mathematical LTP/LTD model was built with the equations *G* = *B*(1−*e^P^* ^/^ *^A^*) + *G*_min_ and *G* = −B(1−*e* ^(^ *^P^* ^−^ *^P^* ^max)/^ *^A^*) + *G*_min_. Figure 4d shows LTP/LTD characteristic curves based on the model in which the nonlinearity of LTP was varied from −16 to 16 and that of LTD was fixed as α_d_ = −16 (*P*_max_ = 64 and Δ*G* = 10). The negative and positive LTP nonlinearity values respectively indicate the synaptic operations of the S‐DNA device and typical ReRAM^23^, ^24^/PCRAM^21^, ^22^ devices. We performed the MNIST simulation once again with various α_p_ values (16, 64, −64, and −16) and then plotted the resulting recognition rates as a function of the learning phase in Figure 4e. Although α_p_ was varied from 16 to −64, the recognition rate after learning with 60 000 MNIST image data did not change much and just varied between 32% and 36%. However, for α_p_ = −16, the recognition rate immediately improved up to ≈42%. This is because the synaptic weights continue to increase as pulse signals for potentiation are supplied, unlike in the case of the conventional LTP characteristic with a positive α_p_ in which synaptic weights are saturated as the number of input pulses increases. In addition, the weight update margin (Δ*G*) also influenced the recognition rate. We prepared LTP/LTD curves with different magnitudes of Δ*G*, with Δ*G* = 1, 10 for the undoped S‐DNA device and Δ*G* = 100 for the Cu^2+^‐doped S‐DNA device (Figure 4f). The following parameters were fixed; α_p_/α_d_ = −16/16, *P*_max_ = 64, and *I*_on_ */I_off_* = 10. Then, by using the three LTP/LTD curves, we performed the MNIST simulation and predicted the recognition rates as a function of the learning phase, as shown in Figure 4g. After completing the MNIST learning process, the recognition rate improved from 40% to 82% when Δ*G* increased from 1 to 100. The negative LTP nonlinearity and high weight update margin influenced the highest and lowest synaptic weights after the full learning process was completed, thereby improving the recognition rate for MNIST patterns.^52^

In conclusion, we studied a salmon deoxyribonucleic acid (S‐DNA)‐based neuromorphic device operating as both a synapse and a neuron. The S‐DNA synaptic and neural operations were based on Cu redox reactions (Cu ↔ Cu^2+^ + 2 e^−^), where the operating mode of the device could be converted by adjusting the duration time (*t*_d_) of the voltage pulse. When a voltage pulse with a short duration time (*t*_d_ = 100 ms) was applied, the device successfully emulated the synaptic function, such as LTP/LTD characteristic. In addition, as applying a voltage pulse with a long duration time (*t*_d_), the device worked as a neuron based on its “integrate‐and‐fire” behavior. In particular, the following remarkable enhancements in the synaptic characteristics were achieved by using the Cu^2+^ doping technique: i) improvement in the nonlinearity of the LTP characteristic (|α_p_|: 31 → 20) and ii) increase in the weight update margin (Δ*G*: 35 → 287 nS). The Cu^2+^ doping also modulated the threshold conditions for the firing of the neuronal device: i) the amplitude of the presynaptic voltage pulse (*V*_pulse_: between 4.5 and 1 V) and ii) the required number of pulses for neuron firing (N: between 8 and 3). This is because Cu^2+^ doping of the S‐DNA electrolyte reduced the activation energy for the formation of the conduction filament. In addition, owing to the negative LTP nonlinearity (the symmetric LTP/LTD characteristic) and the high weight update margin, which were achieved by the Cu^2+^ doping, the MNIST pattern recognition rates improved from 38% to 44% (single‐layer perceptron model) and from 89.42% to 91.61% (multilayer perceptron model) with an increase in the concentration of Cu^2+^ from 0 to 1 × 10^−3^ m.

## Experimental Section

*Preparation of the S‐DNA Solution with Cu^2+^ and an S‐DNA Thin Film*: 0.1 g of S‐DNA (GEM Corporation, Shiga, Japan) was dissolved in 10 mL of deionized (DI) water to make an S‐DNA solution. Then, 0, 0.1, and 1 × 10^−3^ m of Cu^2+^ (i.e., Cu(NO_3_)_2_ purchased from Sigma Aldrich, St. Louis, USA) were added to the S‐DNA solution, followed by magnetic stirring (1000 rpm for 24 h at room temperature). For the formation of an S‐DNA film, 20 µL of the Cu^2+^‐doped S‐DNA solution was spin‐coated on an SiO_2_/Si substrate at 3000 rpm for 90 s, and then the samples were annealed at 100 °C in a vacuum oven for 2 min. The illustration that describes this process is in Figure S1 in the Supporting Information. The thickness of S‐DNA electrolyte was ≈30 nm (see Figure S9, Supporting Information), which was investigated by performing atomic force microscopy (ND‐MDT, NTEGRA Spectra).

*Characterization of the Cu^2+^‐Doped S‐DNA Thin Film*: The S‐DNA thin films with different concentrations of Cu^2+^ (0, 0.1, and 1 × 10^−3^ m) were subjected to Raman spectroscopy (Alpha300 M+, WITec) and FT‐IR (MIR_ATR (ZnSe), Bruker Inc.) analysis. A Raman spectroscope with an excitation wavelength of 532 nm was used. The beam size of the laser was ≈0.7–0.9 µm, and the instrumental spectral resolution was less than 0.9 cm^−1^. The FT‐IR spectral range was from 600 to 3700 cm^−1^, the scan rate was 32 scans per s, and the resolution was ≈4 cm s^−1^. To analyze the FT‐IR data, we subtracted the background spectrum produced by bare glass.

*Fabrication of the S‐DNA Neuromorphic Device*: The surface of the SiO_2_ substrate was cleaned through a sonication process for 10 min in acetone, 2‐propanol, and deionized water. A bottom electrode with Pt (10 nm) and Ti (5 nm) was formed on the SiO_2_ substrate using an e‐beam evaporator and a shadow mask (5‐line pattern, and each line width was 100 µm). After coating a 20 nm thick S‐DNA film on the Pt/Ti/SiO_2_ sample, a top electrode with Cu (30 nm) and Ti (2.5 nm) was deposited using the same shadow mask with the 5‐line pattern.

*Characterization of the S‐DNA Neuromorphic Device*: The fabricated S‐DNA devices were electrically analyzed by using a semiconductor parameter analyzer (HP‐4155A) connected with a voltage pulse generator (Keysight, 33500B). The measurement set up is described in Figure S2 in the Supporting Information. To read the current flowing between pre‐ and postsynaptic terminals, a constant voltage of 0.1 V was applied to a presynaptic terminal. To analyze synaptic characteristics, a series of 15 excitatory/inhibitory pulses (*t*_d_ = 100 ms) was applied to a presynaptic terminal; *V*_pre_ were ± 4.5, ± 1.5, and ± 1 V for the 0, 0.1, and 1 × 10^−3^ m Cu^2+^‐doped S‐DNA devices, respectively. To investigate the neural characteristics of S‐DNA devices, a series of potentiation pulses (*t*_d_ = 500 ms) was used. Here, the values of *V*_pre_ were the same as the values used for synapse characterization.

## Conflict of Interest

The authors declare no conflict of interest.

## Supporting information

Supplementary
