# Supplementary material for: A Neuromorphic Device Implemented on a Salmon‐DNA Electrolyte and its Application to Artificial Neural Networks
Source: Adv Sci (Weinh). 2019 Jul 15;6(17):1901265. doi: 10.1002/advs.201901265 (PMC6724472; doi:10.1002/advs.201901265)
Supplement: Supplementary file 1 — Supplementary [file ADVS-6-1901265-s001.pdf]

Copyright WILEY-VCH Verlag GmbH & Co. KGaA, 69469 Weinheim, Germany, 2019.

ADVANCED SCIENCE
Open Access

Supporting Information

for *Adv. Sci.,* DOI: 10.1002/advs.201901265

A Neuromorphic Device Implemented on a Salmon-DNA Electrolyte and its Application to Artificial Neural Networks

*Dong-Ho Kang, Jeong-Hoon Kim, Seyong Oh, Hyung-Youl Park, Sreekantha Reddy Dugasani, Beom-Seok Kang, Changhwan Choi, Rino Choi, Sungjoo Lee, Sung Ha Park, Keun Heo, and Jin-Hong Park**

Copyright WILEY-VCH Verlag GmbH & Co. KGaA, 69469 Weinheim, Germany, 2019.

Supporting Information

A Neuromorphic Device Implemented on a Salmon-DNA Electrolyte and its Application to Artificial Neural Networks

*Dong-Ho Kang, Jeong-Hoon Kim, Seyong Oh, Hyung-Youl Park, Sreekantha Reddy Dugasani, Beom-Seok Kang, Changhwan Choi, Rino Choi, Sungjoo Lee, Sung Ha Park, Keun Heo, and Jin-Hong Park**

Dr. D.-H. Kang, J.-H. Kim, S. Oh, Dr. H.-Y. Park, B.-S. Kang, Dr. K. Heo, Prof. J.-H. Park
Department of Electrical and Computer Engineering, Sungkyunkwan University, Suwon 16419, Korea
E-mail: jhpark9@skku.edu

Dr. D.-H. Kang
School of Electrical and Electronic Engineering, Nanyang Technological University, 50 Nanyang Avenue, 639798 Singapore, Singapore

Dr. S. R. Reddy, Prof. S. H. Park
Department of Physics, Sungkyunkwan University, Suwon 440-746, South Korea

Prof. C. Choi
Division of Materials Science and Engineering, Hanyang University, Seoul 133-791, South Korea

Prof. R. Choi
Material Science and Engineering, Inha University, Incheon 402-751, South Korea

Prof. J.-H. Park, Prof. S. Lee
SKKU Advanced Institute of Nanotechnology (SAINT), Sungkyunkwan University, Suwon 440-746, South Korea

Formation of Cu^{2+}-doped S-DNA thin film

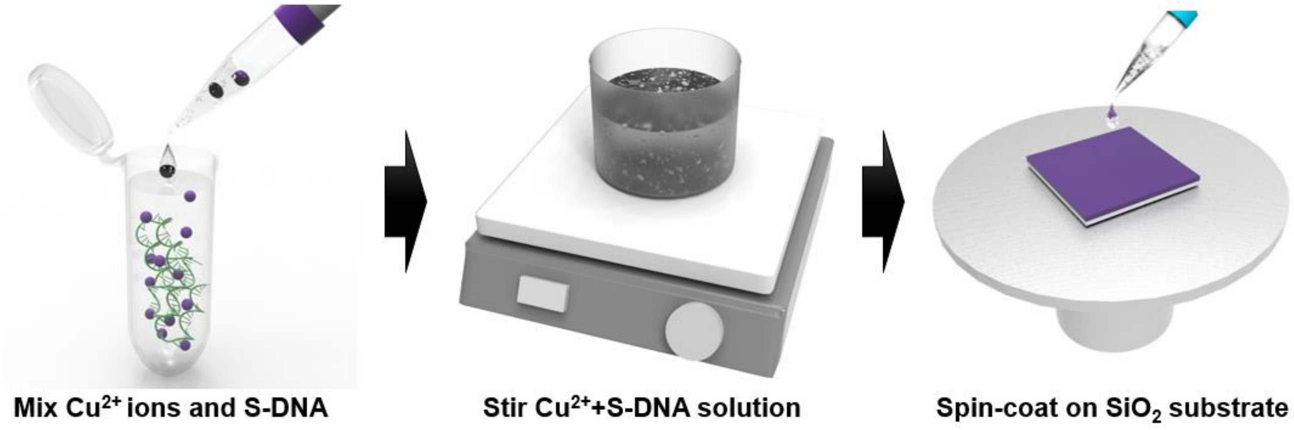

Figure S1. Schematics of the formation of Cu^{2+}-doped S-DNA thin film.

Measurement set-up

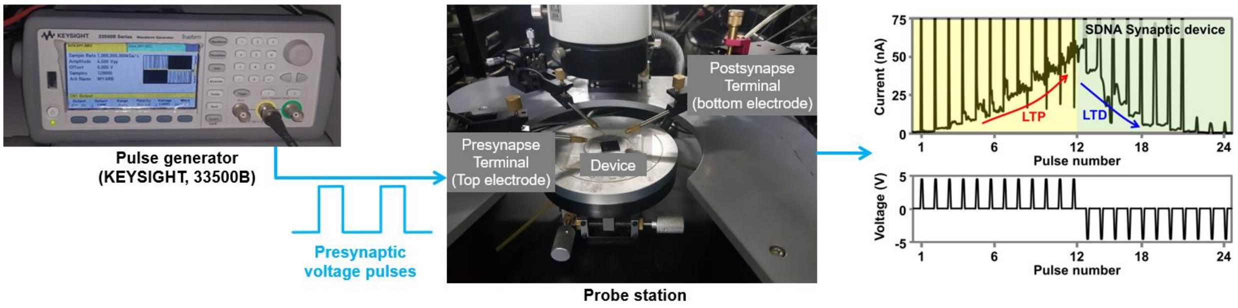

Figure S2. Illustration of the measurement set-up

FT-IR spectra measured on Cu^{2+}-doped S-DNA thin film

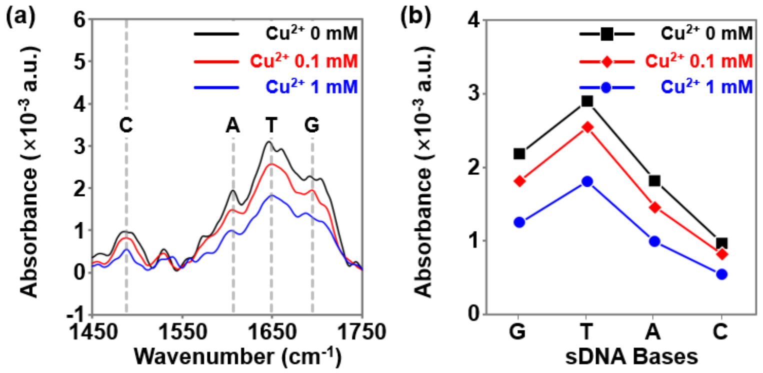

Figure S3. (a) FT-IR spectra and (b) peak intensities for S-DNA base pairs of cytosine (C), adenine (A), thymine (T), and guanine (G).

Figure S3(a) shows the FT-IR spectra measured on the S-DNA thin films doped by 0, 0.1, and 1 mM Cu^{2+}. For the undoped S-DNA film, the FT-IR peaks indicate the S-DNA base pairs of cytosine (C), adenine (A), thymine (T), and guanine (G) observed at 1488, 1603, 1650, and 1695 cm^{-1}, respectively. Here, the peak intensities decreased monotonically with increasing Cu^{2+} concentration. Compared to the undoped S-DNA film, S-DNA films doped with 0.1 and 1 mM Cu^{2+} exhibited approximately 22–31% and 39–50% reduced peak intensities, respectively, as shown in Figure S3(b).

EPSC/IPSC characteristics of 0.1 mM and 1 mM Cu^{2+}-doped S-DNA device

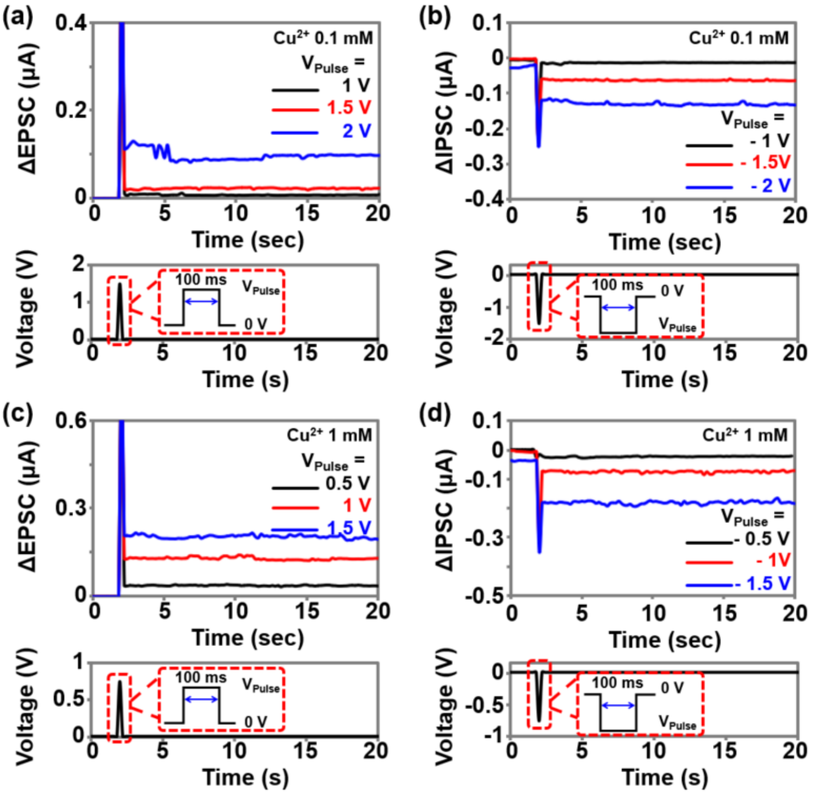

Figure S4. (a) EPSC and (b) IPSC characteristic curves of 0.1 mM Cu^{2+}-doped S-DNA device. (c) EPSC and (d) IPSC characteristic curves of 1 mM Cu^{2+}-doped S-DNA device.

Figure S4 shows the EPSC and IPSC characteristics of 0.1 mM and 1 mM Cu^{2+}-doped S-DNA devices.

i) 0.1 mM Cu^{2+}-doped S-DNA device: when increasing the magnitude of the excitatory pulse from 1 to 2 V, the EPSC value varied from 11.4 to 113.38 nA. Similarly, the ΔIPSC value decreased from –9.04 nA to –100.56 nA when the magnitude of the inhibitory pulse reduced from –1 to –2 V.

ii) 1 mM Cu^{2+}-doped S-DNA device: when increasing the magnitude of the excitatory pulse from 0.5 to 1.5 V, the EPSC value varied from 38.45 to 209.77 nA. Similarly, the ΔIPSC value decreased from –22.26 nA to –218.87 nA when the magnitude of the inhibitory pulse reduced from –0.5 to –1.5 V.

LTP/LTD characteristics of 0 mM, 0.1 mM and 1 mM Cu^{2+}-doped S-DNA device

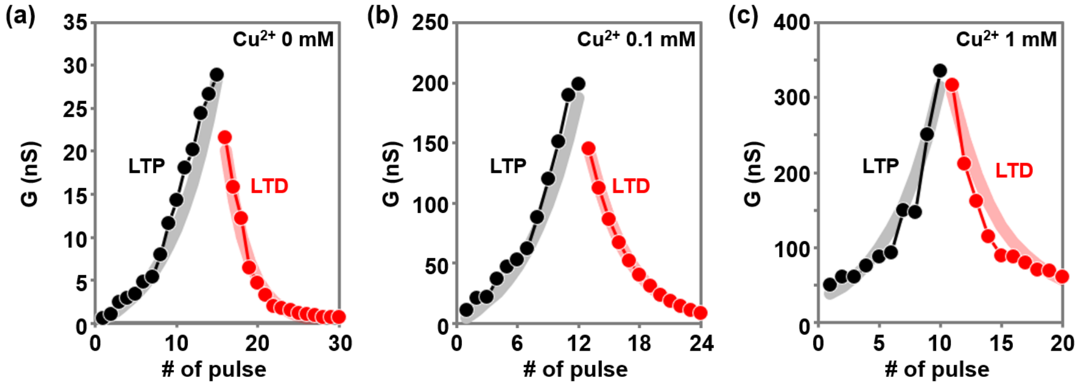

Figure S5. LTP/LTD characteristic data at (a) 0 mM, (b) 0.1 mM, and (c) 1 mM Cu^{2+}-doped S-DNA samples

Learning phase of MNIST simulation.

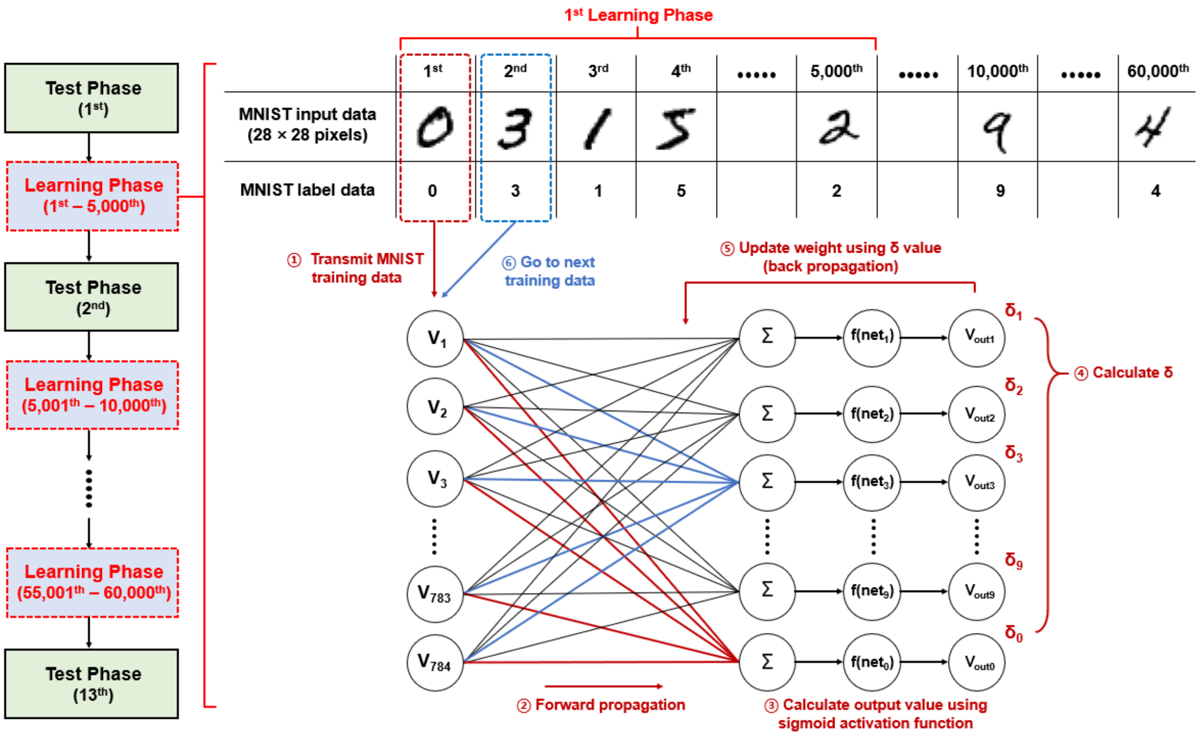

Figure S6. Learning process flow of MNIST simulation.

Figure S6 shows the learning process flow of MNIST simulation. In the learning process, we used a single-perceptron model based on the back-propagation weight updating rule, where the used MNIST learning dataset contains 60,000 hand-written single-digit images (784 pixels) and their representative labels (“0”–“9”). First, each pixel in the learning image was assigned to input neurons as the voltage vector ($\mathbf{V}_1$–$\mathbf{V}_{784}$). The current vector ($\mathbf{I} = \sum \mathbf{V} \times \mathbf{W}$) was then generated at the output neuron by a matrix product of the voltage vector (**V**) and synaptic weight matrix (**W**). This current vector was converted to 10 output values through the sigmoid activation function. Finally, we calculated δ values by subtracting the output values from the labels ($\delta = V_{label} - V_{out}$), which was used to update the synaptic weight. If the sign of the product of δ and the input voltage ($\mathrm{sgn}(\delta \times V_i)$) was positive, the related synapses’ weights increased and vice versa. If the δ value was 0, the synaptic weights were not updated. After updating the synaptic weights, we moved to the next set of learning data.

Test phase of MNIST simulation.

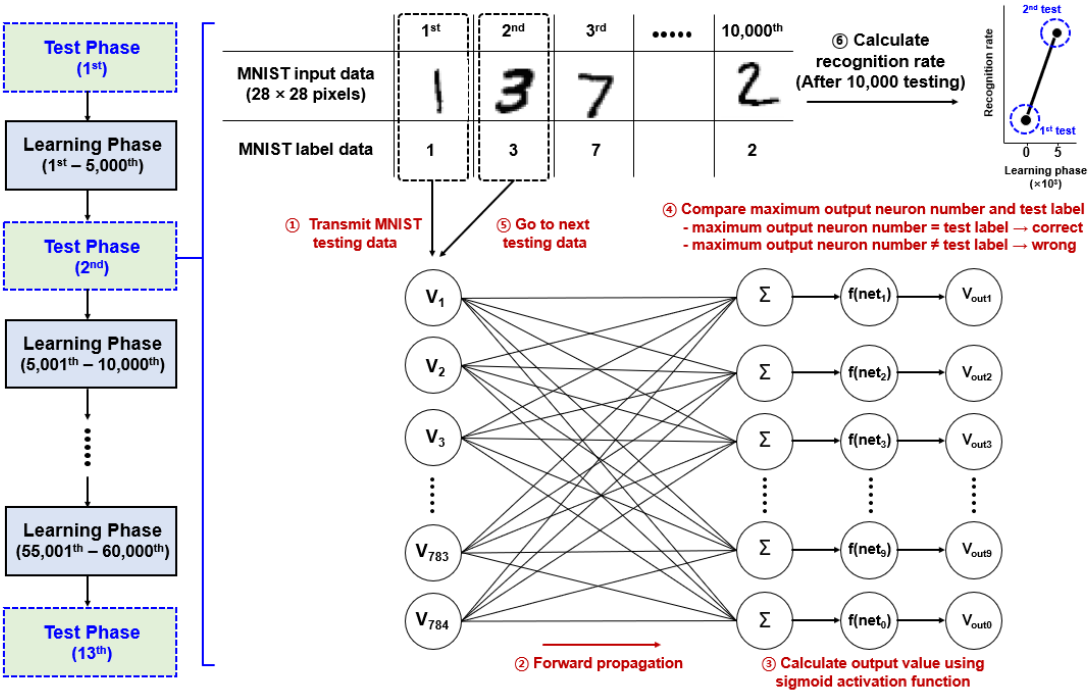

Figure S7. Testing process flow of MNIST pattern simulation.

Figure S7 shows the testing process flow using the MNIST test dataset. This process is used to evaluate how well the trained ANN recognizes MNIST handwritten single-digit images. Thus, the MNIST test dataset, which consists of 10,000 testing images and their representative labels, does not overlap with the learning dataset. From transmitting MNIST testing data to calculating output values, the process flow is the same as that of the learning process. After calculating the output values, we compared the “maximum output neuron number,” the number assigned to the output neuron with the largest output value, with the label data. If they were equal, we judged the inference result as “correct” and vice versa. After testing 10,000 images, we calculated the recognition rate for the ANN by dividing the number of “correct” results by 10,000.

MNIST simulation using a multilayer perceptron-based ANN.

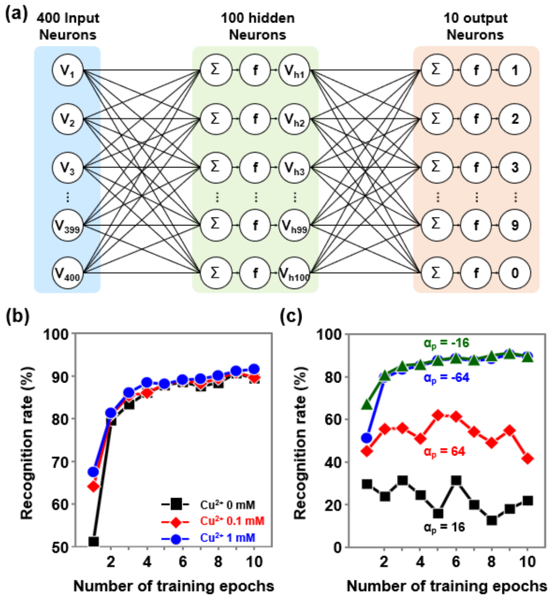

Figure S8. (a) A three-layer perceptron-based artificial neural network. (b) Average recognition rates vs. the number of learning epochs for three cases: 0 mM, 0.1 mM, and 1 mM Cu^{2+}-doped S-DNA. Here, we used 6,000 learning images per each learning epoch. (c) Average recognition rates vs. the number of learning phases for cases where α_p = 16 (black), 64 (red), –64 (blue), and –16 (green).

Figure S8 shows an MNIST simulation that uses a three-layer perceptron-based ANN (400 × 100 × 10) constructed with Cu^{2+}-doped S-DNA devices. The MNIST simulation was conducted on the "+NeuroSim" platform,[S1] which is based on the multilayer perceptron (MLP) model with a stochastic gradient descent weight update. As shown in Figure S6(b), the recognition rate after 60,000 learning phases increased from 89.42% to 91.61% as the Cu^{2+} concentration increased from 0 to 1 mM. In this MLP-based prediction of the recognition rate, we investigated once again how the LTP nonlinearity (α_p) affects the recognition rate. Figure S8(c) shows the MNIST simulation results for various α_p values (16, 64, –64, and –16), where α_d = 16, P_{max} = 64 and ΔG = 10. For $\alpha_p > 0$, the recognition rates after learning with 60,000 MNIST data were lower than 40%. However, as the symmetry of the LTP/LTD characteristics improved (α_p = –16 and –64 for α_d = 16), the recognition rates increased up to approximately 90%, indicating that symmetry is an important parameter that can improve the recognition rate.[S2]

AFM image and depth profile of S-DNA electrolyte.

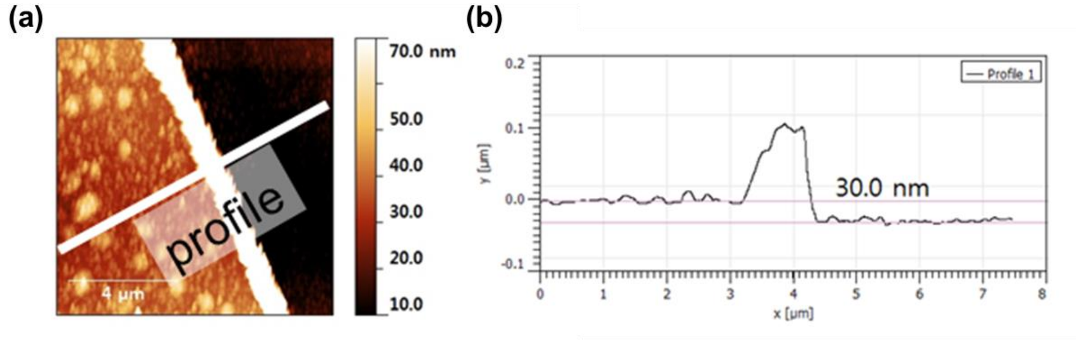

Figure S9. (a) AFM image and (b) depth profile of S-DNA electrolyte. The thickness of S-DNA electrolyte was approximately 30 nm.

Quantitative comparison of MNIST pattern recognition rate.

Synaptic device type	TiO_x/TiO_2 [S3]	PCMO [S4]	Ag:a-Si [S5]	AlO_x/HfO_2 [S6]	FeFET [S2]	S-DNA (This work)	1 mM Cu^{2+}-doped S-DNA (This work)
# of conductance states	102	50	97	40	30	64	64
Nonlinearity	0.66/0.69	3.68/6.76	2.4/4.88	1.94/0.61	–0.6/6	–12/31	–20/18
Dynamic range	2	6.84	12.5	4.43	16.02	47.6	6.71
Weight increase pulse	3 V /40 ms	–2 V /1 ms	3.2 V /0.3 ms	0.9 V /0.1 ms	3.2 V /50 ns	4.5V /100 ms	1.5V /100 ms
Weight decrease pulse	–3 V /10 ms	2 V /1 ms	–2.8 V /0.3 ms	–1 V /0.1 ms	–3.2 V /50 ns	–4.5V /100 ms	–1.5V /100 ms
Online learning accuracy	~10%	~10%	~73%	~41%	~90%	~89%	~91%

Table S1. Benchmarking table obtained on the "+Neurosim" MLP-based platform.

References

[S1] P.-Y. Chen, X. Peng, and S. Yu, *IEEE International Electron Devices Meeting (IEDM)*, 2017, San Francisco, USA.

[S2] M. Jerry *et al.*, *IEEE International Electron Devices Meeting (IEDM)*, 2017, San Francisco, USA.

[S3] L. Gao *et al.*, *Nanotechnology*, 2015, 26, 45, 455204.

[S4] S. Park *et al.*, *IEEE International Electron Devices Meeting (IEDM)*, 2013, Washington, DC, USA.

[S5] S. H. Jo *et al.*, *Nano Lett.*, 2010, 10, 4, 1297-1301.

[S6] J. Woo *et al.*, *IEEE Electron. Dev. Lett.*, 2016, 37, 8, 994-997.
